# Problematic Smartphone Use and Mental Health in Chinese Adults: A Population-Based Study

**DOI:** 10.3390/ijerph17030844

**Published:** 2020-01-29

**Authors:** Ningyuan Guo, Tzu Tsun Luk, Sai Yin Ho, Jung Jae Lee, Chen Shen, John Oliffe, Sophia Siu-Chee Chan, Tai Hing Lam, Man Ping Wang

**Affiliations:** 1 School of Nursing, University of Hong Kong, Hong Kong, China; nyguo@hku.hk (N.G.); luktt@hku.hk (T.T.L.); leejay@hku.hk (J.J.L.); nssophia@hku.hk (S.S.-C.C.); 2 School of Public Health, University of Hong Kong, Hong Kong, China; syho@hku.hk (S.Y.H.); hrmrlth@hku.hk (T.H.L.); 3 Department of Epidemiology and Biostatistics, Imperial College London, London W2 1PG, UK; chen.shen@imperial.ac.uk; 4 School of Nursing, University of British Columbia, Vancouver, BC V6T 2B5, Canada; John.Oliffe@ubc.ca

**Keywords:** problematic smartphone use, smartphone addiction, anxiety, depression, mental well-being, population-based study

## Abstract

Problematic smartphone use (PSU) has been associated with anxiety and depression, but few explored its mental well-being correlates that could co-occur with or be independent of mental symptoms. We studied the associations of PSU with anxiety, depression, and mental well-being in Hong Kong Chinese adults in a probability-based survey (*N* = 4054; 55.0% females; mean age ± SD 48.3 ± 18.3 years). PSU was measured using Smartphone Addiction Scale-Short Version. Anxiety and depression symptoms were evaluated using General Anxiety Disorder screener-2 (GAD-2) and Patient Health Questionnaire-2 (PHQ-2). Mental well-being was measured using Subjective Happiness Scale (SHS) and Short Warwick-Edinburgh Mental Well-Being Scale (SWEMWBS). Multivariable regression analyzed associations adjusting for sociodemographic and lifestyle-related variables. Associations of PSU with mental well-being were stratified by symptom severity of anxiety (GAD-2 cutoff of 3) and depression (PHQ-2 cutoff of 3). We found that PSU was associated with higher odds of anxiety and depression symptom severity and lower scores of SHS and SWEMWBS. Associations of PSU with lower SHS and SWEMWBS scores remained in respondents who screened negative for anxiety or depression symptoms. To conclude, PSU was associated with anxiety, depression, and impaired mental well-being. Associations of PSU with impaired mental well-being could be independent of anxiety or depression symptoms.

## 1. Introduction

The evolving mobile information and communication technologies (ICTs) have raised debates about the potential effects on mental health. Instant messaging (IM) and social networking sites (SNS) can be used for unpleasant moods avoidance, social contacts, and relationship maintenance especially in females [1], whereas a practical and instrumental use such as for social position advancement was found in males [2]. In contrast, many studies have shown the associations between poor mental health outcomes and excessive or intensive use of phone calls, texts, IM, emails, and SNS [3]. These are privileged applications of problematic smartphone use (PSU), an impaired ability to control smartphone use with core symptoms such as loss of control, tolerance, and withdrawal shared with gaming disorder and substance use disorders [4]. PSU has been associated with an array of health outcomes, including self-reported dependence [5], cyberbullying [5], traffic accidence [5], physical symptoms (e.g., eye strain and fatigue) [6], and sleep disturbances [7,8]. Our previous study also showed the lower levels of family communication and well-being associated with PSU [9].

Research in adolescents and young adults have identified psychopathological correlates with PSU, with affective disorders including anxiety and depression were most studied [1]. Small to medium effect size associations were observed between PSU and the severity of anxiety and depression symptoms [10]. Longitudinal studies further supported the predicting effects of PSU on anxiety and depression in college students [7,8]. Although these age groups were deemed to be at higher risk for PSU because of developing self-control and more access to the ICTs, increasing prevalence of both smartphone ownership and PSU in adults of a wider age range warranted general population studies [11,12,13,14].

The definition of mental health has been established as not the mere absence of psychopathological symptoms, which leads researchers to broaden the investigation field to positive psychology [15]. Mental well-being investigates hedonic well-being that includes affective emotions and cognitive assessments of life satisfaction and eudemonic well-being that includes psychological functioning and self-realization [16]. Our previous study showed that people with greater mental well-being tended to have lower risks for anxiety and depression [16]. Despite the correlations, the dual continuum model of mental health proposes that mental illness and well-being are on two distinct dimensions [17]. This notion was supported by studies in adults reporting greater well-being despite concurrent affective disorders or impaired mental well-being but without mental illness [18,19]. One study using data from a sample of college students showed that PSU was negatively correlated with mental well-being outcomes [20]. Little is known about whether such associations could co-occur with or operate independently from affective disorders.

The present study took advantage of a representative population-based survey in Chinese adults in Hong Kong, where the smartphone penetration rate (88.6% in people aged 10 years or above in 2017) has been among the highest worldwide due to advanced cyber-infrastructure and low-cost Internet access [21]. We aimed: (a) to confirm the associations of PSU with anxiety and depression symptom severity in the general population; (b) to examine the associations of PSU with mental health outcomes including hedonic and eudemonic well-being; (c) to examine the associations of PSU with mental well-being outcomes with stratification of symptom severity of anxiety and depression.

## 2. Materials and Methods

### 2.1. Participants and Procedure

The Hong Kong Family and Health Information Trends Survey (FHInTS) is a periodic territory-wide telephone survey on the general public’s behaviors and views regarding information use, individual health, and family well-being, under the project named “FAMILY: A Jockey Club Initiative for a Harmonious Society.” The target population was Cantonese-speaking Hong Kong residents aged 18 years old or above. We have conducted five waves of FHInTS since 2009 and reported details of the study design elsewhere [9,11,16]. The present survey was conducted from February to August 2017, as part of the fifth FHInTS. A two-stage probability-based sampling procedure was used. In the first stage, landline telephone numbers were randomly generated using known prefixes assigned to telecommunication services providers under the numbering plan provided by the Office of the Communication Authority. Invalid numbers were then removed according to the computer and manual dialing records. Numbers of successful cases from previous FHInTS were also filtered. In the second stage, once a household was successfully reached, an eligible family member who would have the nearest next birthday was invited to the survey. Interviews were conducted by trained interviewers from the Public Opinion Programme (POP) at the University of Hong Kong. Of 5773 respondents invited, 4054 were successfully interviewed, yielding a response rate of 70.2%.

### 2.2. Measurements

The ten-item Smartphone Addiction Scale-Short Version (SAS-SV) measured five addiction-like symptoms of PSU, including daily-life disturbance, withdrawal, cyberspace-oriented relationship, overuse, and tolerance [22]. Each item scores on a Likert scale of 1 (strongly disagree) to 6 (strongly agree), with a higher total score (range 10 to 60) indicating a higher PSU level [22]. The Chinese version of SAS-SV was valid and reliable (Cronbach’s alpha 0.84) and indicated acceptable fit for the one-factor model (comparative fit index [CFI] 0.98 [> 0.90 acceptable, > 0.95 excellent]; root mean square error of approximation [RMSEA] 0.08 [< 0.08 acceptable, < 0.05 excellent]; non-normed fit index [NNFI] 0.96 [> 0.95 acceptable]) in our previous study using the same sample of the Hong Kong general population [11]. The convergent validity was adequate, with composite reliability of 0.85 (CR > 0.70 acceptable) and average variance extracted of 0.37 (AVE > 0.40 acceptable if CR > 0.60).

The four-item Patient Health Questionnaire (PHQ-4) includes the two-item General Anxiety Disorder screener (GAD-2) and the two-item Patient Health Questionnaire (PHQ-2) [23]. PHQ-2 has two DSM-IV diagnostic core criteria for major depression disorder, and GAD-2 has two core criteria for generalized anxiety disorder that can also screen for panic and social anxiety disorders [23]. Each item scores on a Likert scale of 0 (not at all) to 3 (nearly every day), with total scores of each subscale ranging 0 to 6 [23]. GAD-2 and PHQ-2 Scores of ≥ 3 are recommended to screen positive for anxiety and depression symptoms, respectively [24]. The Chinese version of PHQ-2 has been validated in our previous study in the Hong Kong general population [25]. In the present sample, confirmatory factor analysis (CFA) indicated an excellent fit for the two-factor model of PHQ-4 (Relative Chi-Square 0.24; *p* = 0.63; incremental fit index [IFI] 1.00 [> 0.95 excellent]; goodness of fit index [GFI] 1.00 [> 0.95 excellent]; adjusted goodness of fit index [AGFI] 1.00 [> 0.95 acceptable]; CFI 1.00; RMSEA 0.01; standardized root mean square residual [SRMR] 0.001 [< 0.08 acceptable, < 0.05 excellent]). GAD-2 had a Cronbach’s alpha of 0.74, CR of 0.65, and AVE of 0.48. PHQ-2 had a Cronbach’s alpha of 0.73, CR of 0.63, and AVE of 0.46.

The four-item Subjective Happiness Scale (SHS; mean score range 1 to 7) measured the cognitive and affective state characterized by pleasure or satisfaction from hedonic well-being aspect [26]. The Chinese version of SHS has been validated in our previous study in the Hong Kong general population [27]. In the present sample, CFA indicated an excellent fit for the one-factor model (relative Chi-Square 21.68; *p* < 0.001; IFI 0.993; GFI 0.999; AGFI 0.995; CFI 0.993; RMSEA 0.10; SRMR 0.02). SHS had a Cronbach’s alpha of 0.75, CR of 0.77, and AVE of 0.47. The seven-item Short Warwick-Edinburgh Mental Well-Being Scale (SWEMWBS; total score range 7 to 35) was adapted by the WEMWBS that covers both hedonic and eudemonic aspects [28]. A randomly selected subset (*n* = 1331, 32.8%) were asked the frequency of feeling optimistic about the future, useful, relaxed, close to other people, dealing with problems well, thinking clearly, making up their own mind about things in past two weeks [28]. The Chinese version of SWEMWBS was valid and reliable (Cronbach’s alpha 0.85) and indicated excellent fit for the one-factor model (CFI 0.995; RMSEA 0.03; SRMR 0.04; normed-fit index [NFI] 0.991 [> 0.95 acceptable]) in our previous study using the same sample of the Hong Kong general population [16]. The convergent validity was adequate, with CR of 0.85 and AVE of 0.44.

Sociodemographic and lifestyle-related variables included sex, age, marital status, employment status, educational attainment, monthly household income, smoking, and alcohol drinking. This list of potential confounders was selected based on prior associations with PSU and mental health outcomes [11,29,30].

### 2.3. Statistical Analysis

We checked the distributions of all variables independently, with a skewness value of ≤ |2.0| and a kurtosis value of ≤ |7.0| indicating the normality [31]. All data were weighted by age, sex, and educational attainment distribution of the Hong Kong general population using the random iterative method [32]. Missing data were handled by available case analyses as there were minimal missing values for all variables (< 2.6%). We examined the Spearman correlations of anxiety and depression with mental well-being outcomes, as scores of GAD-2 and PHQ-2 were not normally distributed. Moderate correlation coefficients (*r*) were observed with scores of SHS (*r* range −0.35 to −0.38; both *p* < 0.001) and SWEMWBS (both *r* = −0.42; both *p* < 0.001). We examined the associations of SAS-SV score with the odds of severity of anxiety and depression symptoms using bivariate and multivariable logistic regression analyses adjusting for sociodemographic and lifestyle-related variables. Bivariate and multivariable linear regression analyses examined the associations of SAS-SV score with scores of SHS and SWEMWBS. We further stratified the associations of SAS-SV score with scores of SHS and SWEMWBS by symptom severity of anxiety (GAD-2 cutoff of 3) and depression (PHQ-2 cutoff of 3). The interaction effects of anxiety and depression symptoms on the associations of SAS-SV score with mental well-being outcomes were examined using adjusted Wald tests. Values of *p* < 0.05 were considered statistically significant. All analyses were conducted using Stata/MP 15.1 (StataCorp LP, College Station, TX, USA), except for CFA that were conducted using LISREL 9.30 with diagonally weighted least square estimation.

### 2.4. Ethics

The Institutional Review Board of the University of Hong Kong/Hospital Authority Hong Kong West Cluster approved this study. All respondents provided verbal informed consent. Telephone interviews were tape-recorded for quality checking with respondents’ consent. Records were then erased six months after completing the survey.

## 3. Results

The weighted sample comprised 55.0% females, and the mean age ± standard deviation (SD) was 48.3 ± 18.3 years (Table 1). Smartphone owners (80.0%) reported a mean SAS-SV score ± SD of 28.9 ± 10.1. Using cutoff points ≥ 3 of GAD-2 and PHQ-2, 10.9% (*n* = 442) and 8.1% (*n* = 327) respondents screened positive for anxiety and depression symptoms, respectively. Mean scores of SHS and SWEMWBS were 5.2 ± 1.0 and 23.0 ± 4.2, respectively.

Higher SAS-SV scores were observed for respondents who screened positive for symptoms of anxiety (31.9 ± 9.4 vs. 28.5 ± 10.2; *p* < 0.001) and depression (33.2 ± 10.2 vs. 28.5 ± 10.0; *p* < 0.001) than those with negative screening results (Table 2). Multivariable analyses showed that a 1-unit increase in SAS-SV score (range 10–60) was associated with a 3% increase in odds of severity of anxiety symptoms (adjusted odds ratio [AOR] = 1.03; 95% CI: 1.01, 1.04) and a 4% increase in odds of severity of depression symptoms (AOR = 1.04; 95% CI: 1.03, 1.06) after adjusting for sociodemographic and health-related variables.

Multivariable analyses showed that a 1-standard-deviation increase in SAS-SV score was associated with a 0.07-standard-deviation decrease in SHS score (adjusted B = −0.07; SE: 0.002; *p* < 0.001) and a 0.10-standard-deviation decrease in SWEMWBS score (adjusted B = -0.10; SE: 0.01; *p* = 0.002) (Table 3). Reduction in effect size of these associations were observed after stratifications by screening results of anxiety and depression symptoms, except for the stronger association of SAS-SV score with lower SHS score in respondents who screened positive for anxiety symptoms (adjusted B = −0.16; SE: 0.01; *p* = 0.013). The associations of SAS-SV score with lower SHS score remained in respondents who screened negative for anxiety symptoms (adjusted B = −0.04; SE: 0.002; *p* = 0.040) and depression symptoms (adjusted B = −0.05; SE: 0.002; *p* = 0.014). The associations of SAS-SV score with lower SWEMWBS score remained in respondents who screened negative for anxiety symptoms (adjusted B = −0.08; SE: 0.01; *p* = 0.022) and were marginally significant in those who screened negative for depression symptoms (adjusted B = −0.06; SE: 0.01; *p* = 0.054). We observed no interaction effects of anxiety or depression symptoms on the associations of SAS-SV score with lower scores of SHS and SWEMWBS.

## 4. Discussion

With a representative sample of Chinese adults in Hong Kong, we confirmed the associations of PSU with anxiety and depression in the general population. Few studies of potential mental health effects of PSU have incorporated both mental illness and mental well−being outcomes. We provided the first evidence of the associations of PSU with impaired hedonic and eudemonic well−being, which remained in respondents who screened negative for anxiety or depression symptoms.

Our study built on young people studies to indicate that the associations of PSU with anxiety and depression could have expanded to adults of all ages. The associations can be explained by the time displacement hypothesis that posits a possible tradeoff between smartphone activities and offline healthier activities such as social interactions [33]. Our previous study supported this explanation by showing that PSU was associated with lower levels of perceived family communication and family well−being [9]. This lack of social support can induce the onset of affective disorders such as anxiety and depression [34]. Other studies showed that PSU symptoms such as overuse and tolerance could risk people to prolong the night−time smartphone usage, which might lead to sleep problems that could mediate the pathway to anxiety and depression [35]. Increasing evidence has suggested that the most problematic application correlated with PSU could be SNS, which could expose people to negative social comparisons with others in perceived more favorable lives and induce affective disorders [36,37,38]. However, people with symptoms of mental illness might be at higher risk for PSU given the smartphone could be the first and most obvious process to deflect negative cognition and affectivity [39]. Mechanisms in this potential reverse causality can include cognitive− and affective−related maladaptive coping strategies such as repetitive negative thinking and emotion dysregulation [39]. The bidirectional association was hence possible and evident by the reciprocal relations found in prospective cohort studies in young people [7,40].

We observed the association of PSU with lower scores of subjective happiness (i.e., hedonic well−being), which is characterized by affectivity of pleasure and cognition of satisfaction [16]. This finding was consistent with studies of Internet addiction with lower levels of happiness and life satisfaction in young people [41,42]. An intervention restricting night−time smartphone usage also reported the reduced PSU risk and increased levels of subjective happiness at one−week follow−up [43]. PSU was associated with lower scores of SWEMWBS that covers both hedonic and eudemonic aspects of mental well−being in the present study. In contrast, a study showed the improved mental well−being in self−concealers who intentionally withhold personal information in face−to−face settings but engaged more in online communication even driven by PSU [44]. These conflicting findings highlighted the important role of personality traits when evaluating the potential effects of PSU and suggested to balance our findings with potential benefits of ICTs usage such as fostering social inclusion among those who may feel excluded [45].

The magnitude of the association of PSU with impaired subjective happiness increased in respondents who screened positive for anxiety symptoms, which suggested the co−occurrence of lower levels of hedonic well−being with anxiety disorder. This finding can be supported by the cognitive−and affective−related coping processes in the pathway from anxiety symptoms to PSU [39]. Another explanation can be the moderate correlation between scores of SHS and GAD−2 found in the present sample. Previous studies also showed that people with symptoms of affective disorders had lower levels of mental well−being than those without [46]. Despite the correlated relations, the independence of mental well−being from mental illness was supported by the remained associations of PSU with mental well−being in respondents who screened negative for anxiety or depression. This finding provided insights that the absence of psychopathological symptoms might have non−buffering effects on the impaired mental well−being outcomes associated with PSU.

Our findings need to be interpreted with caution. Consistent with a systematic review that reported the small effect size associations of PSU with symptom severity of anxiety and depression (adjusted B range 0.12 to 0.18) [10], a 1−unit increase in SAS−SV score was associated with 3%–4% increase in the odds of positive screening results of anxiety and depression in the present study. The small effect size was also observed for the association of PSU with mental well−being outcomes (adjusted B range 0.07 to 0.10). A study across three large−scale datasets (total N = 355358) showed a much smaller association (median adjusted B = −0.07) of adolescents’ digital technologies use with combined mental illness and well−being outcomes [47]. However, unaccounted factors might affect both PSU and mental health in such cross−sectional associations. Longitudinal and experimental studies are warranted to distill causal and predictive models.

One of the study’s limitations is that the cross−sectional data restricts the causal inference of the findings. Residual confounding by unmeasured or unknown confounders might exist even after adjusting for many sociodemographic and lifestyle−related variables. We used the landline telephone survey. Sampling bias might exist due to the lack of data on mobile phone−only households that may have different smartphone use patterns. To increase the sample’s representativeness, we weighted data according to the age, sex, and educational attainment distributions of the Hong Kong general population. We used self−reported data, which are subject to recall bias and social desirability bias. Future studies of PSU could include behavioral methods for collecting data on smartphone use, such as objectively examining participants’ screen time and usage of individual apps. We used screening instruments rather than diagnostic instruments to measure PSU and mental health outcomes. However, more accurate diagnoses by face−to−face assessments in clinical settings would limit the generalizability of findings compared with the population−based study.

## 5. Conclusions

Our population−based study indicated that PSU was associated with higher odds of severity of anxiety and depression symptoms and lower levels of mental well−being. The associations of PSU with impaired mental well−being outcomes could remain in people who screened negative for anxiety or depression symptoms. Such findings highlighted the importance to investigate other psychological constructs with PSU, particularly from aspects of positive psychology. Longitudinal and experimental studies are warranted to explain causality and possible mechanisms of the associations between PSU and mental health.

## Figures and Tables

**Table 1:** Sociodemographic and lifestyle-related characteristics of the sample (*N* = 4054).

Sample Characteristics	Non-Weighted *n* (%)	Weighted ^a^ *n* (%)
**Sex**		
Male	1535 (37.9)	1826 (45.0)
Female	2519 (62.1)	2228 (55.0)
**Age, years**		
18–24	417 (10.3)	370 (9.1)
25–44	573 (14.1)	1436 (35.4)
45–64	1437 35.5)	1498 (37.0)
≥ 65	1627 (40.1)	750 (18.5)
**Marital status**		
Unmarried	852 (21.0)	1164 (28.7)
Cohabitated/married	2577 (63.6)	2478 (61.1)
Divorced/separated/widowed	625 (15.4)	413 (10.2)
**Employment status**		
Unemployment	128 (3.2)	221 (5.5)
In paid employment	1279 (31.6)	1935 (47.7)
Retired	1632 (40.3)	919 (22.7)
Housekeeper	732 (18.1)	722 (17.8)
Full-time student	283 (7.0)	257 (6.3)
**Educational attainment**		
Primary or below	959 (23.7)	959 (23.7)
Secondary	1730 (42.7)	1949 (48.1)
Tertiary	1365 (33.7)	1145 (28.3)
**Monthly household income (HK $ ^b^)**		
≤19999	1671 (41.2)	1465 (36.1)
≥20000	2258 (55.7)	2486 (61.3)
Unsteady	125 (3.1)	103 (2.5)
**Smoking**		
Never	3368 (83.1)	3211 (79.2)
Former	435 (10.7)	467(11.5)
Current	251 (6.2)	376 (9.3)
**Alcohol drinking**		
Never	2065 (51.0)	1908 (47.1)
Former	256 (6.3)	228 (5.6)
Current	1731 (42.7)	1916 (47.3)
**Smartphone ownership**		
No	1126 (27.8)	811 (20.0)
Yes	2928 (72.2)	3243 (80.0)
SAS-SV score (range 10–60; mean ± SD)	28.2 ± 10.0	28.9 ± 10.1
**Anxiety**		
Negative (GAD-2 < 3)	3655 (90.2)	3611 (89.1)
Positive (GAD-2 ≥ 3)	397 (9.8)	442 (10.9)
**Depression**		
Negative (PHQ-2 < 3)	3777 (93.4)	3718 (91.9)
Positive (PHQ-2 ≥ 3)	269 (6.7)	327 (8.1)
**SHS score (range 1–7; mean ± SD)**	5.2 ± 1.0	5.2 ± 1.0
**SWEMWBS score ^c^ (range 7–35; mean ± SD)**	23.4 ± 4.5	23.0 ± 4.2

SAS-SV, Smartphone Addiction Scale-Short Version; GAD-2, Generalized Anxiety Disorder Questionnaire-2 (range 0–6); PHQ-2, Patient Health Questionnaire-2 (range 0–6); SHS, Subjective Happiness Scale; SWEMWBS, Short Warwick-Edinburgh Mental Well-Being Scale. ^a^ Weighted by age, sex, and educational attainment distributions of the Hong Kong general population. ^b^ US $1 = HK $7.8. ^c^ Subset sample (*n* = 1331).

**Table 2:** Odds of anxiety and depression associated with SAS-SV score (*N* = 4054).

Outcomes Associated with SAS-SV Score	SAS-SV Score (Mean ± SD) ^a^	Unadjusted Association		Adjusted ^b^ Association	
		Odds Ratio (95% CI)	*p*	Odds Ratio (95% CI)	*p*
**Anxiety**					
Negative (GAD-2 < 3)	28.5 ± 10.2	1		1	
Positive (GAD-2 ≥ 3)	31.9 ± 9.4	1.03 (1.02, 1.04)	<0.001	1.03 (1.01, 1.04)	<0.001
**Depression**					
Negative (PHQ-2 < 3)	28.5 ± 10.0	1		1	
Positive (PHQ-2 ≥ 3)	33.2 ± 10.2	1.04 (1.03, 1.06)	<0.001	1.04 (1.03, 1.06)	<0.001

SAS-SV, Smartphone Addiction Scale-Short Version (range 10-60); CI, Confidence Interval; GAD-2, Generalized Anxiety Disorder Questionnaire-2 (range 0–6); PHQ-2, Patient Health Questionnaire-2 (range 0–6). ^a^ Weighted by age, sex, and educational attainment distributions of the Hong Kong general population. ^b^ Adjusted for age, sex, marital status, employment status, educational attainment, monthly household income, smoking, and alcohol drinking.

**Table 3:** Standardized beta (B) of SHS and SWEMWBS scores associated with SAS-SV score (*N* = 4054).

Outcomes Associated with SAS-SV Score	Stratification	Unadjusted Association				Adjusted ^a^ Association				*p* for Interaction ^a^
		B (SE)	*p*	F (df = 1)	r^2^	B (SE)	*p*	F (df = 21)	R^2^	
**SHS score (range 1–7)**	**Overall**	−0.08 (0.002)	<0.001	16.99	0.01	−0.07 (0.002)	<0.001	9.26	0.06	−
	**Anxiety**									0.197
	Negative (GAD−2 < 3)	−0.04 (0.002)	0.023	5.15	0.002	−0.04 (0.002)	0.040	7.16	0.06	
	Positive (GAD−2 ≥ 3)	−0.11 (0.01)	0.076	3.17	0.01	−0.16 (0.01)	0.013	0.88	0.07	
	**Depression**									0.626
	Negative (PHQ−2 < 3)	−0.05 (0.002)	0.008	7.13	0.003	−0.05 (0.002)	0.014	7.89	0.06	
	Positive (PHQ−2 ≥ 3)	−0.01 (0.01)	0.864	0.03	0.0002	−0.03 (0.01)	0.707	0.61	0.07	
**SWEMWBS score (range 7–35) ^b^**	**Overall**	−0.11 (0.01)	0.001	10.84	0.01	−0.10 (0.01)	0.002	5.72	0.12	−
	**Anxiety**									0.565
	Negative (GAD−2 < 3)	−0.09 (0.01)	0.014	6.10	0.01	−0.08 (0.01)	0.022	4.54	0.11	
	Positive (GAD−2 ≥ 3)	0.01 (0.03)	0.950	0	0	0.02 (0.04)	0.866	1.00	0.19	
	**Depression**									0.982
	Negative (PHQ−2 < 3)	−0.07 (0.01)	0.028	4.87	0.01	−0.06 (0.01)	0.054	4.85	0.11	
	Positive (PHQ−2 ≥ 3)	−0.03 (0.03)	0.826	0.05	0.001	−0.02 (0.04)	0.895	1.75	0.42	

SAS−SV, Smartphone Addiction Scale−Short Version (range 10–60); SE, Standardized Error; GAD−2, Generalized Anxiety Disorder Questionnaire−2 (range 0–6); PHQ−2, Patient Health Questionnaire−2 (range 0–6); SHS, Subjective Happiness Scale; SWEMWBS, Short Warwick−Edinburgh Mental Well−Being Scale. ^a^ Adjusted for age, sex, marital status, employment status, educational attainment, monthly household income, smoking, and alcohol drinking. ^b^ Subset sample (*n* = 1331).
